# Vegetation-based slope protection using bermuda grass: Effects on the dynamic elastic modulus of weakly expansive soil

**DOI:** 10.1371/journal.pone.0353198

**Published:** 2026-07-10

**Authors:** Xin Huang, Wei Zheng, Li-Dong Tang, Jian-Cai Xie, Chao Chen, Jing-Kai Wu, Hong-Wei Liu, Hui Xu

**Affiliations:** 1 Tonglu County Mineral Resources Management Service Center, Hangzhou, China; 2 School of Civil Engineering and Architecture, Zhejiang Sci-Tech University, Hangzhou, China; 3 Zhejiang Tunnel Engineering Group Company Limited, Hangzhou, China; 4 Zhenan Comprehensive Engineering Surveying and Mapping Institute, Hangzhou, China; 5 Tonglu County Transportation Development Group Company Limited, Hangzhou, China; 6 Zijin School of Geology and Mining, Fuzhou University, Fuzhou, China; China Construction Fourth Engineering Division Corp. Ltd, CHINA

## Abstract

Weakly expansive soil is utilized in subgrades and slope engineering. vegetation is commonly adopted for ecological reinforcement, and its effects on the dynamic mechanical behaviors of weakly expansive soil remain to be further studied. Effects of vegetation, dynamic stress amplitude, confining pressure, and loading frequency on the dynamic strain and dynamic elastic modulus of weakly expansive soil were analyzed in this study. Specifically, different planting densities of Bermuda grass were investigated, including 25 g/m², 40 g/m², 50 g/m², which are denoted as P25, P40 and P50 respectively. Test Results showed that 1) Bermuda grass effectively mitigated the cumulative dynamic strain. Specifically, at *f* = 1 Hz and *σ*_*d*_ = 20 kPa, the cumulative dynamic strain of the P25, P40, and P50 groups decreased by 9.5%, 17.0%, and 19.7% compared with bare soil. 2) Bermuda grass weakened the effect of confining pressure on the dynamic elastic modulus of soil. when *σ*_3_ increased from 50 kPa to 100 kPa, the dynamic elastic modulus of bare soil rose by 16.4%, while the increment of the P50 group was only 1.7%. 3) Bermuda grass also enhanced dynamic stiffness of weakly expansive soil, and the reinforcing effects presented a positive correlation with planting density. Under sinusoidal cyclic loading (*f* = 1 Hz, *σ*_*d*_ = 40 kPa), the dynamic elastic modulus of P25, P40 and P50 were 6.4%,12.8% and 26.16% higher than that those of soil, respectively. Overall, Bermuda grass substantially improves the dynamic deformation resistance and stiffness of weakly expansive soils, which indicates its strong potential for enhancing shear strength and structural stability in expansive soil engineering under dynamic loading.

## 1. Introduction

Expansive soils are widely distributed in more than 60 countries and regions worldwide. In China, the area affected by expansive soils exceeds 107 million square meters, causing economic losses of approximately 90 billion RMB [1,2]. With continuous economic development and increasing land consumption, engineering construction in expansive soil regions is gradually becoming more frequent. Consequently, investigating effective engineering practices in areas with widespread expansive soils has become a significant research focus. With economic development, infrastructures such as highways and railways will be increasingly constructed in areas filled with weakly expansive soils. Consequently, many highways and embankments can only utilize weakly expansive soil as backfill material [3]. However, due to the susceptibility of expansive soil to environmental influences, a series of engineering problems have gradually emerged, including pavement heave, cracking, and slope instability. These issues are largely triggered by dynamic loads (such as vehicular traffic [4]), characterized by high frequency and short duration [5]. The influence of dynamic loading on the shear strength properties of soils is primarily manifested through alterations in soil microstructure [6]. Before shear failure, repeated dynamic loading induces cumulative plastic deformation, continuously changing the original soil structure and reducing its shear strength. Under the same cyclic loading conditions, soils with a higher dynamic elastic modulus tend to exhibit smaller volumetric strain, thereby maintaining greater structural stability. In contrast, soils with a lower dynamic elastic modulus are more prone to significant volumetric deformation, leading to structural destabilization, a reduction in shear strength, and an increased susceptibility to shear failure. Therefore, it is imperative to conduct in-depth investigations into the dynamic response of weakly expansive soils under traffic loading. Numerous studies have explored the potential of vegetation to improve the static mechanical behavior of expansive soils. Studies have demonstrated that plant roots enhance shear strength by increasing soil cohesion through mechanical interlocking, root tensile resistance, and root–soil interface bonding [7,8]. However, most existing studies have focused on static shear reinforcement mechanisms, leaving the dynamic behavior of vegetated expansive soils insufficiently understood.

Previous studies on the dynamic behavior of expansive soils have largely focused on the effects of stabilizing agents. Lv et al. [9] conducted dynamic triaxial tests on rubber-reinforced soils under freezing conditions and found that increasing rubber content gradually flattened the hysteresis loops and reduced the shear modulus. Hou et al. [10] performed dynamic triaxial tests on expansive soils improved with expanded polystyrene and observed that under high confining pressure, loading frequency exerted only a minor influence on the dynamic stress–strain response. Zhuang, Yang and Jin [11] conducted wetting-drying cycles followed by dynamic triaxial tests on remolded expansive soils and found that the dynamic elastic modulus decreased and the damping ratio increased with additional cycles, indicating significant degradation of dynamic characteristics. Yang et al. [12] investigated the effects of freeze–thaw cycles and found that under high confining pressure, the dynamic shear modulus increased after the first freeze–thaw cycle, which differed from the observations under low confining pressure.

Studying the influence of Bermuda grass (*Cynodon dactylon*) on the dynamic elastic modulus of weakly expansive soil provides a novel perspective to better understand how vegetation may influence the shear strength characteristics of such soils under dynamic loading conditions. Laboratory tests were conducted, finding that root systems can substantially alter the dynamic stiffness and damping behavior of fine-grained soils. Shen et al. [13] observed that mixing roots of *Cynodon dactylon* into clay modifies resonant-column responses and the hysteresis behavior under cyclic excitation. Liu et al. [14] showed that different root distribution patterns markedly reduce both resilient and plastic deformations under cyclic triaxial loading, indicating that root can improve dynamic resistance of soil. Xu et al. [15] found that roots of *Cynodon dactylon* inhibit crack propagation and mitigate shear-strength degradation of expansive soils subjected to wetting–drying cycles, suggesting that roots can affect dynamic stiffness. In addition, studies have found that root presence modifies pore structure and hydraulic pathways in compacted soils by affecting microporosity, which may change the way dynamic loads are transmitted and dissipated within the soil mass [16–19].

In this study, dynamic triaxial tests were conducted on both bare soil specimens and root–soil samples obtained from pot tests under cyclic dynamic loading. The effects of dynamic stress amplitude, confining pressure, loading frequency, and the planting densities of Bermuda grass on the dynamic elastic modulus of weakly expansive soils were investigated. This approach allows a clearer understanding of how Bermuda grass influences the shear strength characteristics of weakly expansive soils under dynamic loading. Dynamic stress–strain curves were generated for different dynamic stress amplitudes under various influencing factors, and the corresponding dynamic elastic moduli were calculated. The findings provide a scientific basis for improving engineering performance in regions underlain by weakly expansive soils. These findings clarify vegetation’s dynamic reinforcement of weakly expansive soils under cyclic loads, and offer design references for highway and intercity road cut slopes.

## 2. Materials and methods

### 2.1. Soil and vegetation

The experimental soil was air-dried, passed through a 2 mm sieve, and uniformly moistened with water to achieve the optimum gravimetric water content of 27.5%. The prepared moist soil was sieved again through a 2 mm mesh, placed into the containers, and sealed for 1 d to allow uniform moisture distribution. The index properties of soil are shown in Table 1.

**Table 1:** Index properties of test soil.

Index properties value	Value	Reference
**Unified Soil Classification System (USCS):**	CL	ASTM D2487 [20]
Specific gravity:	2.67	ASTM D854 [21]
The optimum water content (%):	27.5	ASTM D698 [22]
The maximum dry unit density (kg/m^3^):	1472	ASTM D698 [23]
**Atterberg limits**		ASTM D4318 [24]
Liquid limit (%):	45.1	
Plastic limit (%):	28.0	
Plastic Index	17.1	
**Particle size distribution (%)**		ASTM D422 [25]
Sand content (≤2 mm, %):	49.2	
Silt content (≤63 *μ*m, %):	20.1	
Clay content (≤2 *μ*m, %):	30.7	

Bermuda grass was selected for this study owing to its wide applicability in slope and embankment restoration. Bermuda grass seeds were uniformly sown on the soil surface at planting densities of 25 g/m², 40 g/m², and 50 g/m². Two replicate pots were prepared for each density group. The corresponding experimental groups were labeled P25, P40, and P50, while the group without plant roots was labeled as the bare soil group (in Table 2). The plant growth period lasted 47 d, including a 10 d maintenance period and a 37 d active growth period. During the test, each pot received 160 g of water per day.

**Table 2:** Plant characteristic parameters of root–soil specimens.

Test group	Plant density (g/m²)	Root content *R*_*b*_ (kg/m³)	Average shoot length （mm）
P25	25	0.60 ± 0.04	109.5 ± 6.5
P40	40	0.75 ± 0.03	123.6 ± 8.7
P50	50	0.88 ± 0.04	146.87 ± 9.32

During plant growth, the surface of each pot was photographed every 5 d. After the tests, soil samples were randomly collected from selected surface areas of the pots by three-blade samplers (with an inner diameter of 38 mm and a height of 76 mm). The samplers were positioned perpendicular to the pot surface and driven into the soil layer with the aid of a soil extractor. The corresponding volume of root–soil mixtures was then carefully trimmed and removed using a spatula and a soil-cutting knife.

### 2.2. Experimental setup and test procedure

**Fig 1:**
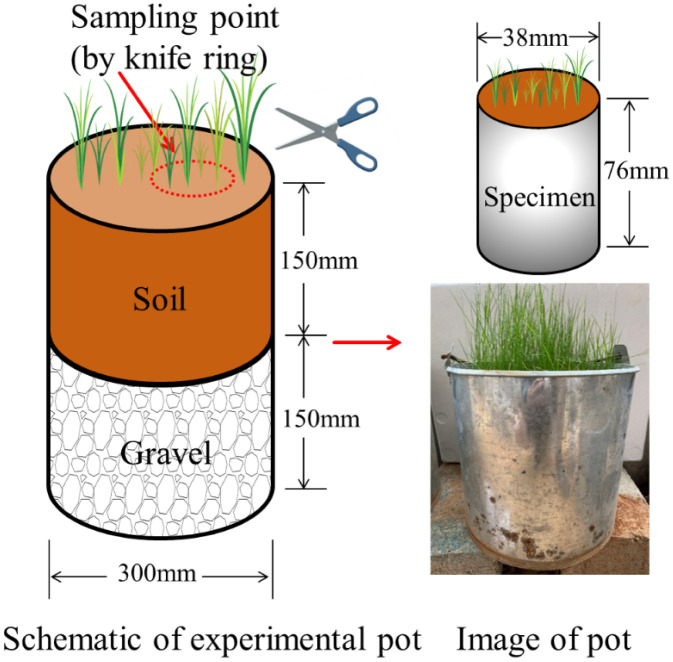
Schematic diagram of the test procedure.

The pot test was conducted using cylindrical containers with an inner diameter of 30 cm and a height of 30 cm, as shown in Fig 1. A drainage hole with a diameter of 1 cm was located at the center of the container bottom to allow excess water to drain, ensuring normal plant growth. The soil was compacted in three layers. First, a 150 mm-thick layer of gravel was placed at the bottom of the container, followed by a layer of geotextile to prevent soil loss. Soil compaction was controlled at 90%, with each layer having a thickness of 5 cm. Before compacting, Vaseline was uniformly applied to the inner walls of the containers to prevent preferential flow.

For soil sampling, a specific area on the pot surface was randomly selected. Containers of different sizes were vertically placed over the selected area, and a soil sampler was pressed into the soil layer. The corresponding volume of root-included soil samples was then carefully extracted using a shovel and a soil cutting tool and the plant characteristics are shown in Table 2

Dynamic triaxial tests in this study were conducted using an ELDyn dynamic triaxial apparatus (Fig 1, Earth Products China Ltd). The dynamic loading module was employed, allowing control of loading frequency, target values, load range, and specimen stiffness. For data acquisition, the system enables the selection of the number of loading cycles, the number of recorded cycles, and the number of data points collected per cycle. The cyclic stress ratio *γ* is defined as the ratio of dynamic stress amplitude to confining pressure [26]:


$$\begin{array}{c} \begin{array}{c} \gamma =\frac{\sigma_{d}}{\sigma_{\text{3}}} \end{array} \end{array}$$ (1)

Dynamic triaxial tests were conducted on both bare soil and rooted soil, with the detailed experimental scheme summarized in Table 3. A series of tests were carried out to examine how confining pressures (i.e., 50 kPa, 100 kPa, and 200 kPa), loading frequencies (i.e., 1 Hz, 2 Hz, and 3 Hz) and dynamic stress amplitudes influence the stress- strain behavior and dynamic elastic modulus of bare and rooted soils. The detailed experimental procedures are descibed as follows:

**Table 3:** Setup of dynamic triaxial test.

Test group	Confining pressure *σ*_3_ (kPa)	Frequency *f* (Hz)	Dynamic stress amplitude (kPa)
1	50	1	20, 30, 40, 50
		2	
		3	
2	100	1	40, 60, 80, 100
		2	
		3	
3	200	1	80, 120, 160, 200
		2	
		3

1) Air inside the apparatus pipelines was evacuated, and a backpressure of 10 kPa was applied. Once no air bubbles were observed in the drainage outlet at the base, the identical degassing procedure was repeated on the other side of the base.
2) A latex membrane was placed on the membrane holder and vacuumed using a suction bulb (specimens were pre-saturated for one at least 1 day before testing). The suction bulb was then released, the specimen placed on the base, and the upper membrane sealed with latex strips.
3) The pressure chamber was installed, and the top vent screw loosened. The sensors were connected to the upper part, after which water was injected until the cap was fully submerged. The cap was then displaced downward until contacting the specimen, and an initial confining pressure of 20 kPa was applied. Air-tightness was checked.
4) After the pressure chamber was installed, the back-pressure valve was opened, and the specimen was further saturated using the back-pressure saturation method.
5) Once saturation was complete, consolidation was carried out in accordance with the target backpressure and confining pressure values defined in the test program. Consolidation was considered complete when no further deformation of the back-pressure system was observed.
6) The dynamic loading module of the control software was activated next. Load control was set according to the experimental scheme, including loading frequency, target dynamic stress, dynamic stress amplitude, and specimen stiffness, with the confining pressure held constant throughout. The cyclic loading test was then initiated.
7) Each dynamic stress amplitude was applied for 10-cycle of cyclic loading before moving on to the next amplitude, adopting the identical 10-cycle loading procedure (for after 8–10 cyclic loads, dynamic elastic modulus of clay stabilizes [27]).
8) The average of the stress-strain peaks from the 2–10 cycles was used to obtain the relationship between dynamic stress amplitude and dynamic strain, from which the dynamic stress–strain curves were plotted.

## 3. Results and discussion

### 3.1. Dynamic stress–strain curve

**Fig 2:**
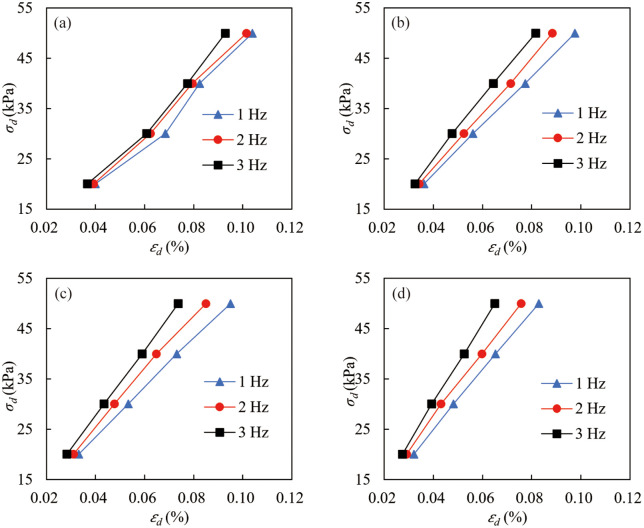
Dynamic stress–strain relationship curve under a confining pressure of 50 kPa. (a) Bare soil (*σ*_3_ = 50 kPa), (b) P25 (*σ*_3_ = 50 kPa), (c) P40 (*σ*_3_ = 50 kPa), and (d) P50 (*σ*_3_ = 50 kPa).

Based on the experimental data, dynamic stress–strain curves were plotted for each test group under three different confining pressures (50 kPa, 100 kPa, and 200 kPa). The dynamic stress–strain curves under a confining pressure of 50 kPa are shown in Fig 2. It can be seen that under a confining pressure of *σ*_3_ = 50 kPa, the bare soil group exhibited the maximum dynamic strain under a dynamic stress amplitude of *σ*_*d*_ = 20 kPa (corresponding to a cyclic stress ratio *γ* of 0.4). Taking the test data at a frequency of *f* = 1 Hz as an example, under *σ*_*d*_ = 20 kPa, the dynamic strain of the P25, P40, and P50 groups decreased by 9.5%, 17.0%, and 19.7%, respectively, compared with the bare soil group. As the dynamic stress amplitude increased, the dynamic strain of all test groups increased corresponding. For instance, at *f* = 1 Hz, the dynamic strain of the bare soil group at the final stress amplitude of *σ*_*d*_ = 50 kPa was 2.60 times that at the initial amplitude of *σ*_*d*_ = 20 kPa. Similarly, the dynamic strains of the P25, P40, and P50 groups at *σ*_*d*_ = 50 kPa were 2.70, 2.86, and 2.58 times their respective initial values recorded at *σ*_*d*_ = 20 kPa.

**Fig 3:**
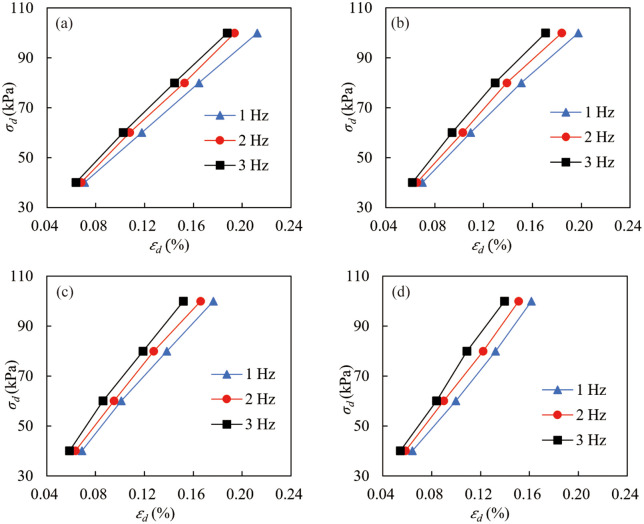
Dynamic stress–strain relationship curve under a confining pressure of 100 kPa. (a) Bare soil (*σ*_3_ = 100 kPa), (b) P25 (*σ*_3_ = 100 kPa), (c) P40 (*σ*_3_ = 100 kPa), and (d) P50 (*σ*_3_ = 100 kPa).

Comparing data at different frequencies and taking *σ*_*d*_ = 50 kPa as an example, the dynamic strain of the bare soil group at *f* = 3 Hz decreased by 10.6% compared with that at *f* = 1 Hz. For the P25, P40, and P50 groups, the reductions were 16.4%, 22.4%, and 21.5%, respectively. The dynamic stress–strain curves under a confining pressure of 100 kPa are shown in Fig 3.

Under a confining pressure of *σ*_3_ = 100 kPa, the bare soil exhibited the largest dynamic strain when the first-level dynamic stress amplitude (*σ*_*d*_ = 40 kPa, *γ* = 0.4) was applied. Taking the data at *f* = 1 Hz as an example, the dynamic strains of the P25, P40, and P50 groups were 1.2%, 2.7%, and 9.3% lower, respectively, than those of the bare soil at the same loading level. As the dynamic stress amplitude increased, the dynamic strain of all test groups rose. At *f* = 1 Hz, the dynamic strain of the bare soil under the final stress amplitude (*σ*_*d*_ = 100 kPa) was 3.00 times that under *σ*_*d*_ = 40 kPa. The corresponding ratios were 2.82 for P25, 2.56 for P40, and 2.51 for P50.

**Fig 4:**
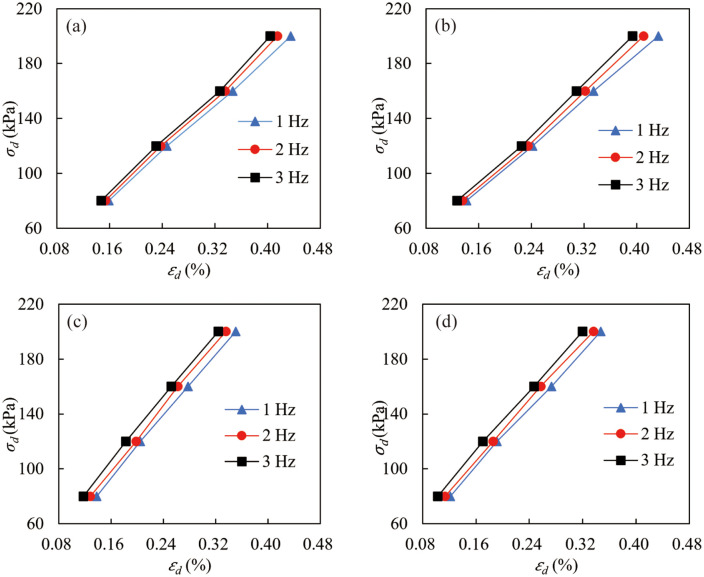
Dynamic stress–strain relationship curve under a confining pressure of 200 kPa. (a) Bare soil (*σ*_3_ = 200 kPa), (b) P25 (*σ*_3_ = 200 kPa), (c) P40 (*σ*_3_ = 200 kPa), and (d) P50 (*σ*_3_ = 200 kPa).

A comparison across different loading frequencies shows similar trends. For *σ*_*d*_ = 100 kPa, the dynamic strain of bare soil at *f* = 3 Hz decreased by 11.6% relative to that at *f* = 1 Hz. Strain reductions of 13.5%, 14.0%, and 13.5% were observed for P25, P40, and P50, respectively. The dynamic stress–strain curves under a confining pressure of 200 kPa are shown in Fig 4.

Under *σ*_3_ = 200 kPa, the P50 group exhibited the smallest dynamic strain when the first-level dynamic stress amplitude (*σ*_*d*_ = 80 kPa, *γ* = 0.4) was applied. Taking *f* = 1 Hz as an example, the dynamic strains of P25, P40, and P50 were 10.9%, 13.0%, and 23.6% lower, respectively, than those of the bare soil at the same loading level. As the dynamic stress amplitude increased, all groups exhibited rising dynamic strain. At *f* = 1 Hz, the dynamic strain of the bare soil under *σ*_*d*_ = 200 kPa was 2.74 times that under *σ*_*d*_ = 80 kPa. The corresponding ratios were 3.06, 2.54, and 2.86 for P25, P40, and P50, respectively. For *σ*_*d*_ = 200 kPa, increasing the frequency from 1 Hz to 3 Hz resulted in dynamic strain reductions of 7.1%, 9.0%, 7.6%, and 7.8% for the bare soil, P25, P40, and P50 groups, respectively.

These results indicate that higher planting density and increased root content effectively reduce the dynamic strain of weakly expansive soil under cyclic loading, contributing to improved structural stability and greater soil strength. A comparison between the effects of Bermuda grass and loading frequency shows that, under *σ*_3_ = 50 kPa and *σ*_3_ = 200 kPa, the strain-reduction effect of frequency is weaker than that induced by Bermuda grass. This difference likely arises from their distinct mechanisms: Bermuda grass roots reduce pore volume, enhance structural stability, and increase shear strength, whereas a higher loading frequency restricts the deformation capacity of the soil during cyclic loading. Although recoverable deformation decreases, the soil structure may already be disturbed, leading to a reduction in shear strength.

### 3.2. Effect of dynamic stress amplitude on the dynamic elastic modulus

The dynamic elastic modulus $E_{d}$ is defined as the slope of the line connecting the two endpoints of the hysteresis loop, representing the soil’s capacity to resist deformation under cyclic loading. $E_{d}$ can be calculated as follows:


$$\begin{array}{c} \begin{array}{c} E_{d}=\frac{\sigma_{b}-\sigma_{a}}{\varepsilon_{b}-\varepsilon_{a}} \end{array} \end{array}$$ (2)

where *σ*_b_ and *σ*_a_ denote the maximum and minimum dynamic stresses on the hysteresis loop, and *ε*_*b*_ and *ε*_*a*_ are the corresponding maximum and minimum dynamic strains. A larger dynamic elastic modulus indicates a smaller strain under the same dynamic loading, implying a more stable soil structure and a higher shear strength.

**Fig 5:**
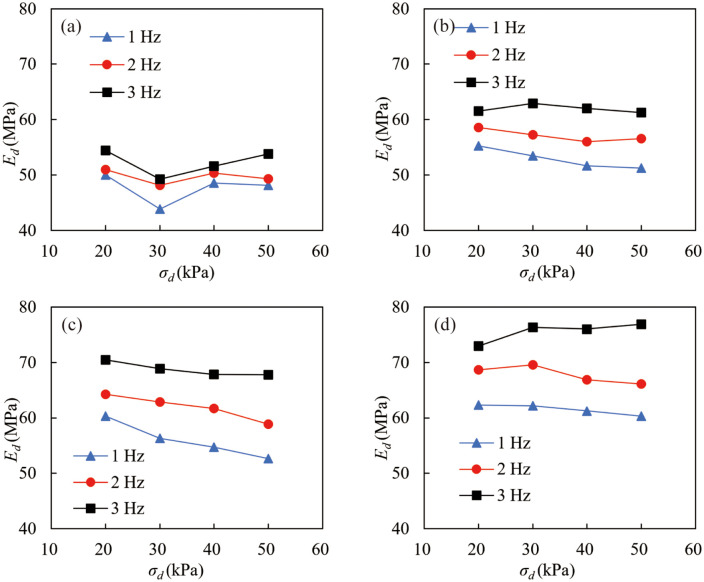
Dynamic elastic modulus–dynamic stress amplitude relationship under a confining pressure of 50 kPa. (a) Bare soil (*σ*_3_ = 50 kPa), (b) P25 (*σ*_3_ = 50 kPa), (c) P40 (*σ*_3_ = 50 kPa), and (d) P50 (*σ*_3_ = 50 kPa).

Based on the calculated dynamic elastic modulus (by Eq. (2)), the dynamic elastic modulus–dynamic stress amplitude relationships for each test group under different confining pressures were plotted. The curves under a confining pressure of 50 kPa are shown in Fig 5. Under *σ*_3_ = 50 kPa, the dynamic elastic modulus exhibited no obvious increasing or decreasing trend with increasing dynamic stress amplitude, but rather fluctuated within a certain range. Taking the data at *f* = 3 Hz as an example, the maximum dynamic elastic modulus of bare soil was 9.4% lower than its minimum value, and the corresponding fluctuation ranges were 2.6% for P25, 3.8% for P40, and 5.1% for P50.

**Fig 6:**
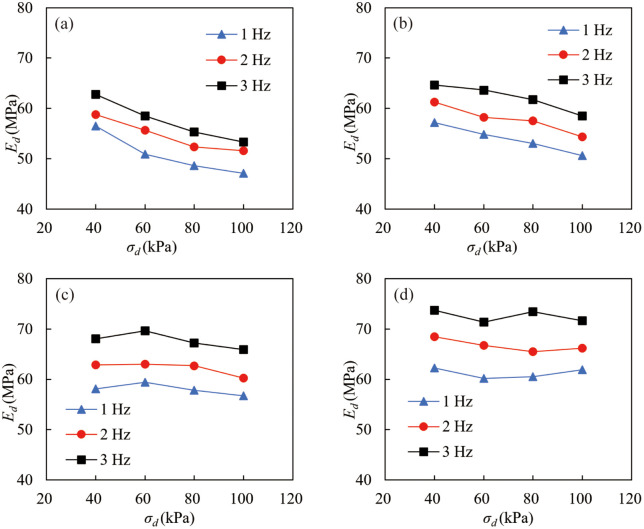
Dynamic elastic modulus–dynamic stress amplitude relationship under a confining pressure of 100 kPa. (a) Bare soil (*σ*_3_ = 100 kPa), (b) P25 (*σ*_3_ = 100 kPa), (c) P40 (*σ*_3_ = 100 kPa), and (d) P50 (*σ*_3_ = 100 kPa).

The dynamic elastic modulus–dynamic stress amplitude curves under *σ*_3_ = 100 kPa are shown in Fig 6. Under this confining pressure, no distinct trend was observed for the P40 and P50 groups as the dynamic stress amplitude increased. For instance, at *f* = 3 Hz, the maximum dynamic elastic modulus decreased by 5.4% for P40 and by 3.2% for P50. In contrast, the bare soil and P25 groups exhibited a more pronounced reduction with increasing dynamic stress amplitude. At *f* = 3 Hz, the maximum dynamic elastic modulus decreased by 15.1% for the bare soil and by 9.5% for P25. Notably, as planting density increased, the influence of confining pressure on the dynamic elastic modulus diminishes (in the P50 group at *f* = 3 Hz, *E*_*d*_ even exhibited an inflection point as *σ*_*d*_ increased). This occurs because plant roots enhance the spatial confinement of soil, increase interfacial friction energy dissipation, elevate equivalent cohesion, and stabilize microstructures. Consequently, the root-soil composite develops stronger disturbance resistance under dynamic loading [13].

**Fig 7:**
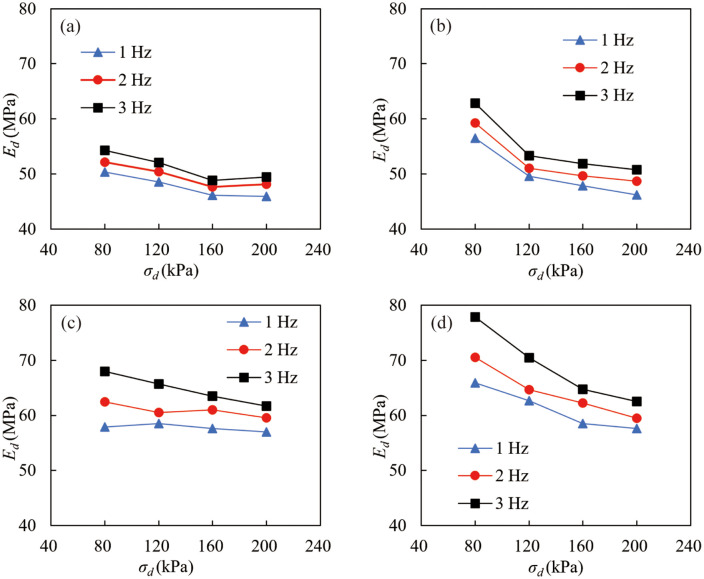
Dynamic elastic modulus–dynamic stress amplitude relationship under a confining pressure of 200 kPa. (a) Bare soil (*σ*_3_ = 200 kPa), (b) P25 (*σ*_3_ = 200 kPa), (c) P40 (*σ*_3_ = 200 kPa), and (d) P50 (*σ*_3_ = 200 kPa).

The curves under *σ*_3_ = 200 kPa are presented in Fig 7. Under this confining pressure, all test groups showed a clear decreasing trend in dynamic elastic modulus with increasing dynamic stress amplitude. At *f* = 3 Hz, the maximum dynamic elastic modulus decreased by 10.1% for the bare soil, 19.2% for P25, 9.3% for P40, and 19.7% for P50.

Overall, under *σ*_3_ = 200 kPa, all test groups exhibited a consistent decrease in dynamic elastic modulus as the dynamic stress amplitude increased. Under *σ*_3_ = 100 kPa, this decreasing trend appeared only in the bare soil and P25 groups. When the confining pressure was further reduced to 50 kPa, no clear pattern was observed for any test group. According to Yang et al. [28], increasing the dynamic stress amplitude causes the cumulative plastic strain of expansive soil to grow rapidly with the number of loading cycles, resulting in a rapid reduction in dynamic elastic modulus. The fact that the P40 and P50 groups exhibited a decreasing modulus only under *σ*_3_ = 200 kPa may be attributed to the variation of macropore volume. Under lower confining pressures, changes in dynamic elastic modulus are mainly governed by variations in macropore volume. The P40 and P50 groups contain fewer macropores than the bare soil and P25 groups. This may be attributed to the fact that the Bermuda grass root system reduces the porosity of the weakly expansive soil, resulting in a more stable soil structure and an increase in shear strength [29]. In contrast, an increase in loading frequency prevents the specimen from fully deforming under dynamic loading. Although the recoverable deformation decreases during testing, the structural damage already induced within the soil reduces its shear strength [30].

### 3.3. Effect of confining pressure on the dynamic elastic modulus

**Fig 8:**
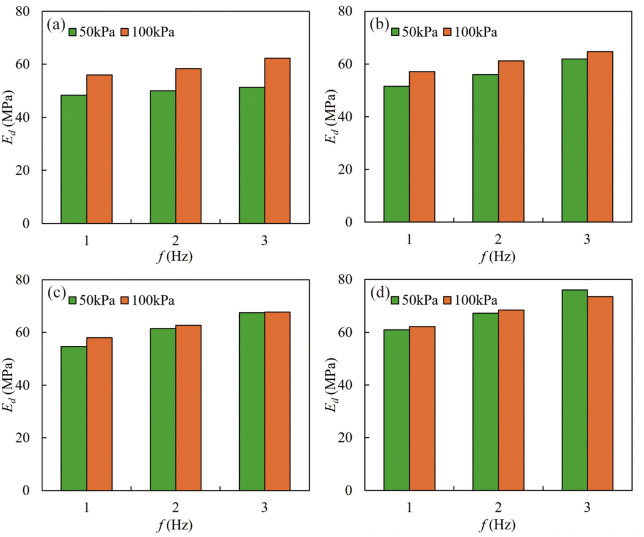
Relationship between dynamic elastic modulus, loading frequencies and confining pressure under a dynamic stress amplitude of 40 kPa. (a) Bare soil, (b) P25, (c) P40, and (d) P50.

To examine the effect of confining pressure on the dynamic elastic modulus, test data obtained under different confining pressures but identical dynamic stress amplitudes of 40 kPa and 80 kPa were compared, and the corresponding dynamic elastic modulus versus confining pressure and loading frequency were plotted. The relationships at a dynamic stress amplitude of 40 kPa are shown in Fig 8. Under *σ*_*d*_ = 40 kPa, the dynamic elastic modulus of the bare soil and P25 groups increased significantly with increasing confining pressure, whereas the P40 and P50 groups exhibited only slight variations. Taking *f* = 1 Hz as an example, the dynamic elastic modulus under *σ*_3_ = 100 kPa increased by 16.4% for the bare soil, 10.7% for P25, 6.1% for P40, and 1.7% for P50 compared with the corresponding values under *σ*_3_ = 50 kPa.

**Fig 9:**
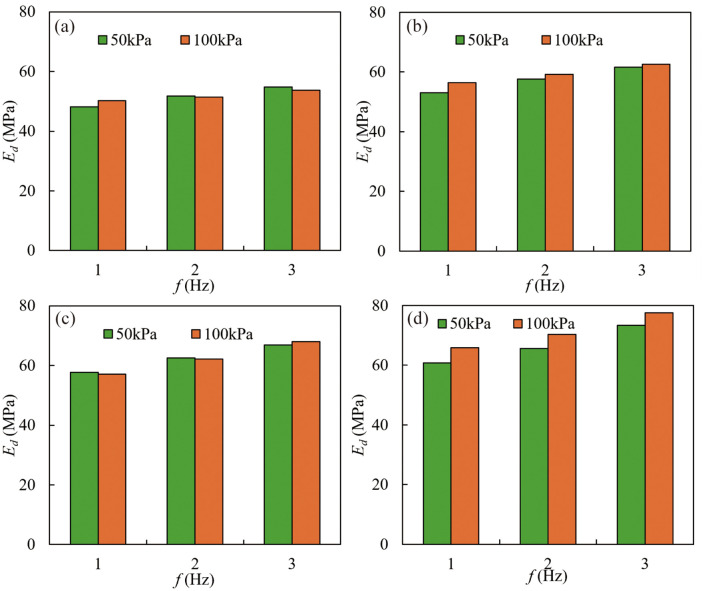
Relationship between dynamic elastic modulus, loading frequencies and confining pressure under a dynamic stress amplitude of 80 kPa. (a) Bare soil, (b) P25, (c) P40, and (d) P50.

The relationship between dynamic elastic modulus, confining pressure and loading frequencies under *σ*_*d*_ = 80 kPa is shown in Fig 9. Under this loading condition, the P50 group exhibited the largest increase in dynamic elastic modulus as confining pressure increased. At *f* = 1 Hz, the dynamic elastic modulus of the P40 group remained nearly unchanged between the two confining pressures, while the modulus values under *σ*_3_ = 200 kPa increased by 3.6% for the bare soil, 6.5% for P25, and 9.0% for P50 compared with those under *σ*_3_ = 100 kPa.

Overall, under lower confining pressures, the dynamic elastic moduli of bare soil and P25 groups increased as confining pressure increased. Under higher confining pressures, this increasing trend became more pronounced for the P50 group. Leong and Cheng [31] reported that an increase in confining pressure strengthens particle–particle interactions within the soil, thereby suppressing deformation and increasing the dynamic elastic modulus. At low confining pressures, deformation of soil is strongly influenced by the volume of macropores. The bare soil and P25 groups had larger macropore volumes than P40 and P50 groups. As confining pressure increased, soil became compressed, resulting in reduced differences in macropore volume among the groups. In this stage, the reinforcing effect of Bermuda grass roots enhances particle interlocking within the root–soil composite, thereby increasing its shear strength. Higher shear strength contributes to structural stability under dynamic loading and reduces volumetric strain of soil [14]. Consequently, the dynamic elastic modulus of the P50 group increased significantly, indicating that dynamic elastic modulus and shear strength are mutually influential.

Previous studies [32–34] have shown that an increase in dynamic elastic modulus effectively enhances the stability of soil structure by reducing volumetric deformation under dynamic loading, thereby contributing to higher shear strength. Conversely, an increase in shear strength allows the soil to undergo smaller volumetric deformation under dynamic loads, which in turn contributes to an increase in dynamic elastic modulus.

### 3.4. Effect of loading frequency on the dynamic elastic modulus

**Fig 10:**
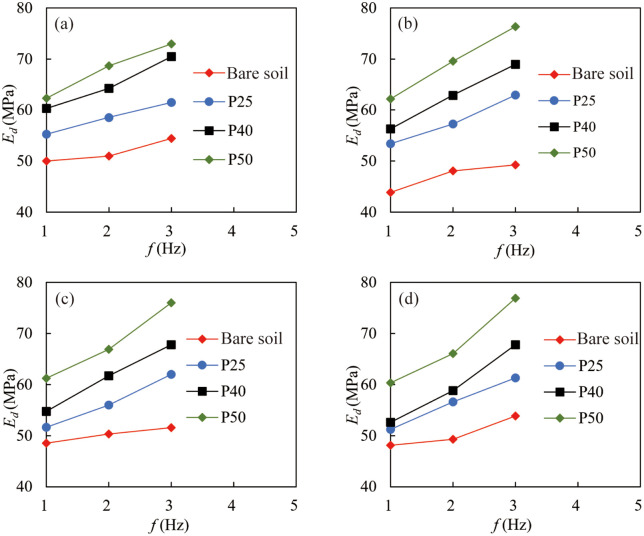
Dynamic elastic modulus–frequency relationship curves. (a) *σ*_*d*_ = 20 kPa, (b) *σ*_*d*_ = 30 kPa, (c) *σ*_*d*_ = 40 kPa, and (d) *σ*_*d*_ = 50 kPa.

To investigate the effect of loading frequency on the dynamic elastic modulus of weakly expansive soil, the data obtained at a confining pressure of *σ*_3_ = 50 kPa were analyzed, and the dynamic elastic modulus–frequency curves for each dynamic stress amplitude were plotted, as shown in Fig 10. It can be observed that the dynamic elastic modulus of both bare soil and rooted soil samples increased with increasing frequency. Taking the data at a dynamic stress amplitude of *σ*_*d*_ = 40 kPa as an example, the dynamic elastic modulus at *f* = 3 Hz increased by 6.3% for the bare soil, 20.1% for P25, 23.9% for P40, and 24.1% for P50 compared with the values at *f* = 1 Hz. Rakshanda et al. [35] reported that at low frequencies, soil deformation is more complete and particle–particle interactions are reduced. Therefore, soil strength is higher under high-frequency conditions.

### 3.5. Effect of Bermuda grass on the dynamic elastic modulus

**Fig 11:**
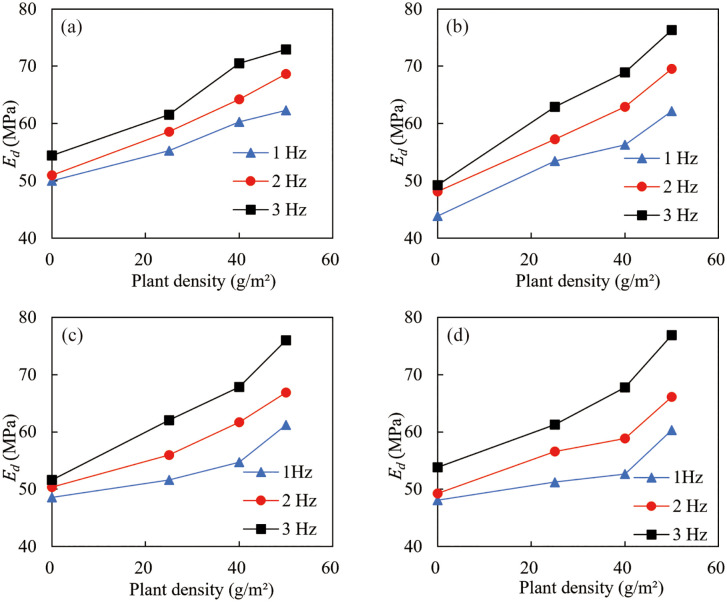
Dynamic elastic modulus–planting density relationship curves. (a) *σ*_*d*_ = 20 kPa, (b) *σ*_*d*_ = 30 kPa, (c) *σ*_*d*_ = 40 kPa, and (d) *σ*_*d*_ = 50 kPa.

After analyzing the effects of dynamic stress amplitude, confining pressure, and loading frequency on the dynamic elastic modulus of weakly expansive soil, the influence of Bermuda grass on the dynamic elastic modulus was further examined. Taking a confining pressure of *σ*_3_ = 50 kPa as an example, the dynamic elastic moduli of all test groups at a frequency of 2 Hz was plotted against planting density, as shown in Fig 11.

It can be observed that the dynamic elastic modulus of weakly expansive soil increases with increasing planting density. For instance, at *f* = 1 Hz and *σ*_*d*_ = 40 kPa, the dynamic elastic modulus of P25 was 6.4% higher than that of bare soil, while P40 and P50 showed increases of 12.8% and 26.2% relative to P25, respectively. Liu et al. [14] conducted dynamic triaxial tests on silty clay at different dynamic stress amplitudes and found that root reinforcement was most pronounced under small dynamic stress (15 kPa), resulting in the greatest increase in dynamic strength. Therefore, the reinforcing effect of plant roots can reduce dynamic strain under cyclic loading, thereby increasing the dynamic elastic modulus. Moreover, plant roots can reduce the macropore volume in soil, limiting the maximum total strain that can develop under the same dynamic stress amplitude [36], which further increases the dynamic elastic modulus. The interconnected root systems inside the soil matrix produce a prominent reinforcement effect, which can effectively constrain the plastic deformation of soil under cyclic dynamic loading. The mechanical interlocking interaction at the root-soil interface also strengthens the bonding between soil particles and roots, improving the overall stiffness of the rooted soil. Compared with the effect of loading frequency on the dynamic elastic modulus, the improvement caused by plant roots is more significant.

A summary of all influencing factors indicates that under high confining pressures, the dynamic elastic modulus clearly decreases with increasing dynamic stress amplitude. Confining pressure exerts a more pronounced influence under low-pressure conditions, significantly increasing the moduli of bare soil and P25, yet its impact is less obvious for the other test groups except for a moderate increase in P50 under high confining pressure. Higer loading frequency, greater planting density and larger root content all enhance the dynamic elastic modulus. Comparing these factors, Bermuda grass exerts a stronger effect on increasing the modulus, which is consistent with finding of Shen et al. [13].

In summary, Bermuda grass not only enhances the shear strength of weakly expansive soils but also reduces strain induced by dynamic loading. This is consistent with previous research findings, namely that under dynamic loading, Bermuda grass primarily influences soil shear strength through two mechanisms: increasing the inherent shear strength of soil [37] and mitigating dynamic strain [13]. Combined, these effects preserve the structural stability of weakly expansive soils and improve their resistance to shear failure.

## 4. Conclusions

Based on the shear strength of bare soil and rooted soil specimens, dynamic triaxial tests were conducted to investigate the influence of Bermuda grass on the shear strength characteristics of weakly expansive soil under dynamic loading. Dynamic stress–strain curves and dynamic elastic moduli of different test groups were derived from test data, and the effects of dynamic stress amplitude, confining pressure, loading frequency, and vegetation on the dynamic stress–strain behavior and dynamic elastic modulus were analyzed. The main findings of this study are summarized as follows:

1) Observations of the dynamic stress–strain curves indicate that dynamic strain increases with increasing dynamic stress amplitude. With increasing frequency, the dynamic strain of weakly expansive soil decreases, with rooted soil samples being more sensitive to frequency. Under high confining pressures, the difference in dynamic strain between different frequencies is relatively small. Comparing bare soil and rooted soil under the same conditions, the dynamic strain of rooted soil is smaller, and dynamic strain further decreases with increasing planting density and root content.
2) Based on the calculated dynamic elastic modulus, it was found that under high confining pressure, the dynamic elastic modulus of all test groups decreases noticeably with increasing dynamic stress amplitude. The effect of confining pressure on the modulus is mainly observed under low confining pressures, where increasing pressure significantly enhances the modulus of bare soil and P25, and under high confining pressure, a slight increase is observed in P50. Increasing frequency, planting density, and root content all contribute to higher dynamic elastic modulus; however, the reinforcing effect of Bermuda grass has a more pronounced impact.
3) Bermuda grass not only improves the shear strength of weakly expansive soil but also reduces the dynamic strain during cyclic loading, enhancing the soil’s ability to resist shear failure under dynamic loading conditions. It offers an effective solution for weak expansive soil slopes exposed to long-term traffic dynamic loading.

## Supporting information

S1 File Support Information 0617. (XLSX)
